# An Anti-Interference Demultiplexing Method for Electromagnetic Bessel Beams Carrying Orbital Angular Momentum

**DOI:** 10.3390/s25216706

**Published:** 2025-11-02

**Authors:** Congwei Mi, Xiuqiong Huang, Wensheng Qiao, Yanming Zhang

**Affiliations:** 1 National Key Laboratory of Complex Aviation System Simulation, Southwest China Institute of Electronic Technology, Chengdu 610036, China; 2 Department of Electronic Engineering, The Chinese University of Hong Kong, Hong Kong, China; ymzhang@cuhk.edu.hk

**Keywords:** electromagnetic bessel beams, orbital angular momentum, smoothed dynamic mode decomposition, anti-interference signal processing, partial aperture detection

## Abstract

This work presents a simple yet effective anti-interference demultiplexing method for electromagnetic Bessel beams carrying orbital angular momentum (OAM), based on smoothed dynamic mode decomposition (smoothed DMD). The method combines conventional dynamic mode decomposition (DMD) with a moving average pre-processing step to enhance its noise resilience. By modeling the azimuthally sampled field as a spatial–temporal signal, smoothed DMD enables accurate extraction of OAM topological charges even under low signal-to-noise ratio (SNR) conditions. Numerical results demonstrate its superior anti-interference performance compared to standard DMD. Moreover, the proposed approach is applicable to scenarios with partial aperture detection and does not rely on the orthogonality of OAM modes, making it particularly suitable for real-world, imperfect conditions. This method offers a robust solution for OAM beam analysis in next-generation wireless communication and sensing applications.

## 1. Introduction

Electromagnetic (EM) waves carrying orbital angular momentum (OAM) possess a helical phase structure characterized by an azimuthal phase term $e^{jl\phi }$, where *l* is known as the topological charge [1,2,3]. This topological property gives rise to a theoretically unbounded set of orthogonal modes, which can be exploited as an additional degree of freedom in both radio frequency (RF) [4,5] and optical communication systems [6]. Leveraging this spatial multiplexing capability, OAM has emerged as a promising solution to meet the growing demand for high-capacity, spectrum-efficient communication in next-generation wireless and optical networks [7,8]. However, despite the theoretical advantages, the practical implementation of OAM-based multiplexing faces several critical challenges [9,10,11,12]. Among them, noise-induced interference during mode demultiplexing remains a major obstacle, especially in real-world propagation environments where the ideal orthogonality of OAM modes is no longer preserved [6,9,13,14]. In particular, random disturbances such as additive white Gaussian noise (AWGN), scattering, or receiver aperture truncation can distort the spatial structure of the beam and lead to power leakage across adjacent modes [9,13]. This results in mode crosstalk, erroneous mode classification, and, ultimately, degraded system performance [6,14].

In RF applications, AWGN is often used to model the background noise originating from environmental and electronic sources [13,15]. When OAM beams propagate through such a noisy medium, the azimuthal phase gradients, which are critical for accurate topological charge identification, become irregular and unreliable [16]. Traditional demultiplexing techniques such as Fourier-based mode decomposition or spatial matched filtering are vulnerable to these distortions [17,18]. Their performance degrades sharply with decreasing signal-to-noise ratio (SNR), particularly when only partial field information is available due to limited aperture size or misalignment at the receiver end [19].

In this paper, we propose a smoothed dynamic mode decomposition (smoothed DMD) framework as a simple yet effective signal processing tool for anti-interference demultiplexing of OAM-carrying Bessel beams. Our approach is based on two key principles: (1) interpreting the azimuthal samples of the field as a discrete spatial–temporal signal, and (2) applying a moving average filter to suppress noise before conducting modal analysis via dynamic mode decomposition (DMD). DMD is a data-driven algorithm that extracts dominant spatial modes and their associated eigenfrequencies from sequentially sampled data, without requiring explicit channel models or assumptions of orthogonality [20,21,22]. By augmenting DMD with a lightweight pre-smoothing step, our method, namely smoothed DMD, achieves greater robustness to noise while preserving the modal features required for topological charge identification. Various numerical results validate the superiority of smoothed DMD over conventional DMD, especially in scenarios with high noise levels and limited aperture. The proposed method not only improves the accuracy of OAM mode demultiplexing but also relaxes the need for strict mode orthogonality and full-aperture measurements, making it highly applicable to practical communication and sensing systems using OAM beams.

The remainder of this paper is organized as follows: Section 2 introduces the mathematical formulation of the Bessel beam and describes the smoothed DMD’s application to Bessel beam decomposition. Section 3 presents simulation experiments under varying noise and aperture conditions to evaluate performance. Section 4 concludes this work.

## 2. Methods

### 2.1. Scalar and Vector Bessel Beam

For scalar Bessel beams in free space and source-free regions, the scalar field $E(\vec{r},t)$ satisfies the wave equation:

(1) $$\nabla^{2}E\left(\vec{r},t\right)-\frac{1}{c^{2}}\frac{\partial^{2}}{\partial t^{2}}E\left(\vec{r},t\right)=0$$

where $\nabla^{2}$ denotes the Laplacian, *c* is the speed of light in vacuum, and $\vec{r}$ is the position vector. Assuming a time-harmonic solution of the form $E\left(\vec{r},t\right)=\Phi \left(\vec{r}\right)e^{-j\omega t}$, substituting into the above yields the scalar Helmholtz equation:

(2) $$\nabla^{2}\Phi \left(\vec{r}\right)+k^{2}\Phi \left(\vec{r}\right)=0$$

where $k=\omega \sqrt{\mu_{0}\varepsilon_{0}}$ is the wavenumber in free space. Applying separation of variables in cylindrical coordinates (ρ,φ,z) leads to a solution involving Bessel functions in the radial direction and Fourier modes in the angular direction. The general form of the solution is

(3) $$E\left(\vec{r},t\right)=E_{0}J_{n}\left(k_{⊥}\rho \right)\exp\left(jn\varphi \right)\exp\left(j\left(k_{z}z-\omega t\right)\right)$$

where $E_{0}$ is a constant amplitude, $J_{n}$ is the *n*th-order Bessel function of the first kind, and $k_{⊥}^{2}+k_{z}^{2}=k^{2}$. The cylindrical coordinates relate to Cartesian coordinates via $\rho =\sqrt{x^{2}+y^{2}}$, x=ρcosφ, and y=ρsinφ. Clearly, Equation (3) represents a scalar Bessel beam of order *n*. This beam is characterized by a transverse profile governed by the Bessel function $J_{n}\left(k_{⊥}\rho \right)$, which is non-diffracting and exhibits a central intensity null when n≠0. The term $e^{jn\varphi }$ denotes an azimuthal phase dependence, indicating the presence of orbital angular momentum (OAM) with topological charge *n*. This scalar solution provides an essential foundation for analyzing vector Bessel beams, which describe realistic electromagnetic fields. In the following, we extend this framework by introducing vector potentials and solving the vector Helmholtz equation to obtain the full electric and magnetic field components corresponding to transverse electric and transverse magnetic Bessel beam modes.

For a vector Bessel beam in source-free free space, elimination of the magnetic field from Maxwell’s equations leads to the wave equation for the electric field [23]:

(4) $$\nabla \times \nabla \times \vec{E}=-\mu_{0}\varepsilon_{0}\frac{\partial^{2}}{\partial t^{2}}\vec{E}$$

Using the vector identity $\nabla \times \left(\nabla \times \vec{E}\right)=\nabla \left(\nabla \cdot \vec{E}\right)-\nabla^{2}\vec{E}$ and the divergence-free condition $\nabla \cdot \vec{E}=0$, we obtain the vector Helmholtz equation:

(5) $$\nabla^{2}\vec{E}+k^{2}\vec{E}=0$$

where $k^{2}=\omega^{2}\mu_{0}\varepsilon_{0}$. In spherical coordinates, a common approach is to use the ansatz $\vec{E}=\vec{r}\times \left(\nabla \psi \right)$, thereby reducing the problem to solving the scalar Helmholtz equation for ψ. To construct solutions that satisfy Maxwell’s equations, we employ the electric and magnetic Hertz vector potentials, denoted as ${\vec{\prod }}_{e}$ and ${\vec{\prod }}_{m}$, respectively. The corresponding electric and magnetic fields are expressed as [24,25]:

(6) $$\begin{array}{cc} {\vec{E}}_{e} & =\nabla \times \nabla \times {\vec{\prod }}_{e}=\nabla \nabla \cdot {\vec{\prod }}_{e}+k^{2}{\vec{\prod }}_{e}\\ {\vec{H}}_{e} & =j\omega \varepsilon_{0}\nabla \times {\vec{\prod }}_{e}\\ {\vec{E}}_{m} & =-j\omega \mu_{0}\nabla \times {\vec{\prod }}_{m}\\ {\vec{H}}_{m} & =\nabla \times \nabla \times {\vec{\prod }}_{m}=\nabla \nabla \cdot {\vec{\prod }}_{m}+k^{2}{\vec{\prod }}_{m} \end{array}$$

Under the source-free assumption, both ${\vec{\prod }}_{e}$ and ${\vec{\prod }}_{m}$ satisfy the homogeneous vector Helmholtz equation [23]:

(7) $$\begin{array}{c} \nabla^{2}{\vec{\prod }}_{e}+k^{2}{\vec{\prod }}_{e}=0\\ \nabla^{2}{\vec{\prod }}_{m}+k^{2}{\vec{\prod }}_{m}=0 \end{array}$$

We adopt the simplification ${\vec{\prod }}_{e}=\prod_{e}\vec{z}$ and ${\vec{\prod }}_{m}=\prod_{m}\vec{z}$, thereby reducing the vector Helmholtz equations to scalar forms [26]:

(8) $$\begin{array}{c} \nabla^{2}\prod_{e}+k^{2}\prod_{e}=0\\ \nabla^{2}\prod_{m}+k^{2}\prod_{m}=0 \end{array}$$

Following the separable solution approach, both scalar potentials $\prod_{e}$ and $\prod_{m}$ can be written as [26]:

(9) $$\prod_{e,m}=P_{e,m}J_{n}\left(k_{⊥}\rho \right)\exp\left(in\varphi \right)\exp\left(j\left(k_{z}z-\omega t\right)\right)$$

where $J_{n}\left(\cdot \right)$ denotes the *n*th-order Bessel function of the first kind, $k_{⊥}$ is the transverse wave number, $k_{z}$ is the longitudinal wave number, and $P_{e,m}$ are the magnitudes of the electric and magnetic dipole moments, respectively. Then, ${\vec{\prod }}_{e}$ and ${\vec{\prod }}_{m}$ can be expressed in the form

(10) $$\begin{array}{cc} \; & {\vec{\prod }}_{e}=\prod_{e}\vec{z}\\ \; & {\vec{\prod }}_{m}=\prod_{m}\vec{z} \end{array}$$

Substituting these expressions into the field definitions yields explicit forms for the transverse magnetic (TM_*n*_) and transverse electric (TE_*n*_) modes.

For the TM_*n*_ mode, one can obtain [24]

(11) $$\begin{array}{cc} E_{\rho e} & =jP_{e}k_{⊥}k_{z}J_{n}^{\prime }\left(k_{⊥}\rho \right)\exp\left(jn\varphi \right)\exp\left[j\left(k_{z}z-\omega t\right)\right]\\ E_{\varphi e} & =-\frac{n}{\rho }P_{e}k_{z}J_{n}\left(k_{⊥}\rho \right)\exp\left(jn\varphi \right)\exp\left[j\left(k_{z}z-\omega t\right)\right]\\ E_{ze} & =P_{e}k_{⊥}^{2}J_{n}\left(k_{⊥}\rho \right)\exp\left(jn\varphi \right)\exp\left[j\left(k_{z}z-\omega t\right)\right]\\ H_{\rho e} & =\frac{n}{\rho }P_{e}\omega \varepsilon J_{n}\left(k_{⊥}\rho \right)\exp\left(jn\varphi \right)\exp\left[j\left(k_{z}z-\omega t\right)\right]\\ H_{\varphi e} & =jP_{e}k_{⊥}\omega \varepsilon J_{n}^{\prime }\left(k_{⊥}\rho \right)\exp\left(jn\varphi \right)\exp\left[j\left(k_{z}z-\omega t\right)\right]\\ H_{ze} & =0 \end{array}$$

For the TE_*n*_ mode, one can obtain [24]

(12) $$\begin{array}{cc} \; & E_{\rho m}=-\frac{n}{\rho }P_{m}\omega \mu J_{n}\left(k_{⊥}\rho \right)\exp\left(jn\varphi \right)\exp\left[j\left(k_{z}z-\omega t\right)\right]\\ \; & E_{\varphi m}=-jP_{m}k_{⊥}\omega \mu J_{n}^{\prime }\left(k_{⊥}\rho \right)\exp\left(jn\varphi \right)\exp\left[j\left(k_{z}z-\omega t\right)\right]\\ \; & E_{zm}=0\\ \; & H_{\rho m}=jP_{m}k_{⊥}k_{z}J_{n}^{\prime }\left(k_{⊥}\rho \right)\exp\left(jn\varphi \right)\exp\left[j\left(k_{z}z-\omega t\right)\right]\\ \; & H_{\varphi m}=-\frac{n}{\rho }P_{m}k_{z}J_{n}\left(k_{⊥}\rho \right)\exp\left(jn\varphi \right)\exp\left[j\left(k_{z}z-\omega t\right)\right]\\ \; & H_{zm}=P_{m}k_{⊥}^{2}J_{n}\left(k_{⊥}\rho \right)\exp\left(jn\varphi \right)\exp\left[j\left(k_{z}z-\omega t\right)\right] \end{array}$$

From the above field expressions, it is evident that OAM is encoded in the azimuthal phase dependence $e^{in\varphi }$, which appears in multiple field components across both TM and TE modes. For instance, each nonzero $E_{\rho }$, $E_{\varphi }$, or $E_{z}$ component in the TM mode inherently carries OAM with an azimuthal index *n*, reflecting the vector Bessel beam’s structured wavefront.

### 2.2. Smoothed Dynamic Mode Decomposition

Bessel beams carrying OAM exhibit a characteristic helical phase profile on the transverse plane. The electric field can be modeled as a superposition of multiple OAM modes:

(13) $$B\left(\rho ,\phi \right)=\sum_{l=1}^{L}R_{l}\left(\rho \right)e^{jn_{l}\phi }$$

where $n_{l}$ denotes the topological charge of the *l*th mode, and $R_{l}\left(\rho \right)$ is its radial amplitude profile. This representation is applicable to scalar beams as well as co-polarized components of vector fields. In particular, vector Bessel beams, such as the TM modes, exhibit OAM content in all field components ($E_{\rho }$, $E_{\phi }$, $E_{z}$) and can be treated accordingly.

To estimate the unknown topological charges $n_{l}$ from field measurements, we apply dynamic mode decomposition (DMD), a data-driven technique originally developed to identify coherent structures in fluid dynamics [27]. DMD is especially well-suited to our case due to the mathematical similarity between the spatial–angular harmonic term $e^{jn\phi }$ and the temporal harmonic $e^{j\omega t}$ traditionally used in DMD applications. Thus, the azimuthal sampling of Bessel beams can be treated as a spatial analogue of time-series data.

Let the field be sampled at *M* uniformly spaced azimuthal angles $\phi_{0},\phi_{1},\phi_{2},\ldots ,\phi_{T}$ with a fixed radial vector ρ.

(14) $$B=\left[\begin{array}{ccccc} b_{0} & \cdots & b_{t} & \cdots & b_{T} \end{array}\right]\in C^{P\times (T+1)},$$

For scalar fields or flattened vector components, we construct two data matrices:

(15) $$\begin{array}{ccc} B_{1} & =\left[\begin{array}{ccccc} b_{0} & \cdots & b_{t} & \cdots & b_{T-1} \end{array}\right] & \in C^{P\times T}, \end{array}$$

(16) $$\begin{array}{ccc} B_{2} & =\left[\begin{array}{ccccc} b_{1} & \cdots & b_{t} & \cdots & b_{T} \end{array}\right] & \in C^{P\times T}. \end{array}$$

Based on the linear assumption between two adjacent states, i.e., $b_{t+1}=Fb_{t}$, the relationship between Equations (15) and (16) can be expressed as $B_{2}=FB_{1}$. Herein, F refers to the mapping matrix, and the purpose of DMD is to calculate the leading eigenvalues and eigenvectors of F. Singular value decomposition (SVD) is used to implement the DMD steps. Initially, we apply the SVD to the first matrix $B_{1}$, which is expressed [8]

(17) $$B_{1}=USV^{*},$$

where * denotes the conjugate transpose. By substituting Equation (17) into $B_{2}=FB_{1}$, we can derive the mapping matrix F as [8]

(18) $$F=B_{2}VS^{-1}U^{*}.$$

Then, U is employed to project the original system into a corresponding reduced-dimensional system. Specifically, the original snapshots are transformed using the proper orthogonal decomposition (POD) basis. It can be represented as ${\hat{B}}_{1}=U^{*}B_{1}\;\;\mathrm{and}\;\;{\hat{B}}_{2}=U^{*}B_{2}$. In this reduced-dimensional system, indicated by the notation ($\hat{\cdot }$), we define the transformation matrix $\hat{F}=U^{*}\mathrm{FU}$. Consequently, we can express the mapping relationship in the reduced-dimensional system by [8]:

(19) $${\hat{B}}_{2}=\hat{F}{\hat{B}}_{1}.$$

Importantly, the mapping matrix $\hat{F}$ in the reduced-dimensional system effectively captures dynamic characteristics. We then address the eigenvalue problem in this compressed framework by performing an eigendecomposition of $\hat{F}$:

(20) $$\hat{F}D=D\Lambda ,$$

where D consists of the eigenvectors of $\hat{F}$, and Λ, a diagonal matrix, contains the eigenvalues, denoted as $\lambda_{k}$ for k=1,2,…,K. Leveraging these results, we define the DMD eigenvectors in the original system as follows [28]:

(21) $$G=B_{2}VS^{-1}D.$$

The field at a given phase can be represented as [8,28]:

(22) $$b\left(\phi_{t}\right)=\sum_{m=1}^{M}g_{m}a_{m}e^{j\omega_{m}\phi_{t}}.$$

Here, $g_{m}$ is the *m*-th column of the matrix G, and $\omega_{m}=\mathrm{real}\left(\omega_{m}\right)+j_{0}\mathrm{imag}\left(\omega_{m}\right)=\ln\left(\lambda_{m}\right)/\Delta \phi$. By comparing the DMD expression of the data, namely Equation (22), to a standard signal model, namely Equation (13), we can deduce DMD allows us to extract the topological charges $n_{1},n_{2},\ldots ,n_{L}$ directly from measured field data.

However, in realistic scenarios, the measured fields are corrupted by Gaussian white noise, which degrades the eigenvalue estimation and, consequently, the accuracy of topological charge extraction. To mitigate this, we introduce a moving average pre-processing step before applying DMD. This technique enhances robustness by reducing noise while preserving OAM-related structural patterns. To perform averaging every η columns, we define the smoothed state ${\tilde{b}}_{k}$ as:

(23) $${\tilde{b}}_{k}=\frac{1}{\eta }\sum_{i=0}^{\eta -1}b\left(\phi_{k}+\eta \Delta \phi \right)$$

Then, the smoothed matrix is given by:

(24) $$\tilde{B}=\left[{\tilde{b}}_{0},{\tilde{b}}_{1},\ldots ,{\tilde{b}}_{K-\eta }\right]$$

Due to the zero-mean property of Gaussian noise, the variance is reduced by a factor of η:

(25) $$\mathrm{Variance}\;\mathrm{of}\;\mathrm{noise}=\frac{\sigma^{2}}{\eta }.$$

We now integrate the moving average into the DMD framework. Let the smoothed matrices be:

(26) $${\tilde{B}}_{1}=\left[{\tilde{b}}_{0},{\tilde{b}}_{1},\ldots ,{\tilde{b}}_{K-\eta -1}\right],$$

(27) $${\tilde{B}}_{2}=\left[{\tilde{b}}_{1},{\tilde{b}}_{2},\ldots ,{\tilde{b}}_{K-\eta }\right],$$

These matrices replace $B_{1}$ and $B_{2}$ in the DMD pipeline. We first perform SVD on ${\tilde{B}}_{1}$

(28) $${\tilde{B}}_{1}=\tilde{U}\tilde{S}{\tilde{V}}^{*}$$

and compute the smoothed mapping matrix as:

(29) $$\tilde{F}={\tilde{B}}_{2}\tilde{V}{\tilde{S}}^{-1}{\tilde{U}}^{*}.$$

Similarly, we perform the eigendecomposition of $\tilde{F}$ to construct the dynamic modes and corresponding eigenvalues. Finally, the expression of the smoothed state can be obtained by

(30) $$\tilde{b}\left(\phi_{k}\right)=\sum_{m=1}^{M}{\tilde{g}}_{m}{\tilde{a}}_{m}e^{j{\tilde{\omega }}_{m}\phi_{k}}.$$

Hence, ${\tilde{\omega }}_{m}$ implies the topological charges of the input field. It is clear that this smoothed DMD method improves the robustness of topological charge extraction from noisy Bessel beam measurements by enhancing the signal-to-noise ratio, enabling reliable demultiplexing of multiple coexisting OAM modes. The proposed smoothed DMD method assumes synchronous arrival of all OAM modes. If a time delay exists between modes, the method is not directly applicable. And the approach could be extended to three-dimensional spatial–temporal OAM beams to address such scenarios in future work. It is worth noting that the proposed smoothed DMD-based decomposition method is theoretically applicable to Bessel beams carrying orbital angular momentum at any wavelength, as long as the sampled field data satisfy the spatial and temporal resolution requirements. It should be noted that the proposed smoothed DMD method assumes zero-mean noise characteristics. Hence, it remains effective for suppressing random Gaussian-type distortions, but its performance may degrade under non-zero-mean or highly irregular noise conditions.

## 3. Results

In this section, we evaluate the performance of the proposed smoothed DMD method for demultiplexing Bessel beams carrying OAM. We first investigate the baseline performance on a single OAM mode, and then progressively increase the number of superposed Bessel beams. The accuracy of topological charge extraction and the robustness to Gaussian noise are assessed under each condition.

### 3.1. Single Bessel Beam

We first evaluate the demultiplexing performance when a single OAM mode is present. Four topological charges are tested: n=1,2,3,4. For each case, the smoothed DMD is applied to extract the dominant eigenvalue, from which the corresponding frequency ω and the topological charge estimate $\hat{n}$ are obtained. We first evaluate the demultiplexing performance when a single OAM mode is present. Four topological charges are tested: n=1,2,3,4. For each case, the proposed smoothed DMD method is applied to extract the dominant eigenvalue, from which the angular frequency $\tilde{\omega }$ and the estimated topological charge *n* are obtained.

**Figure 1:**
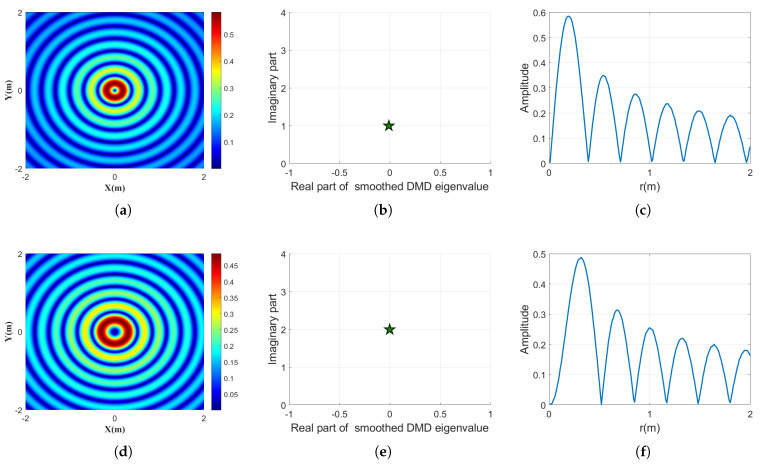

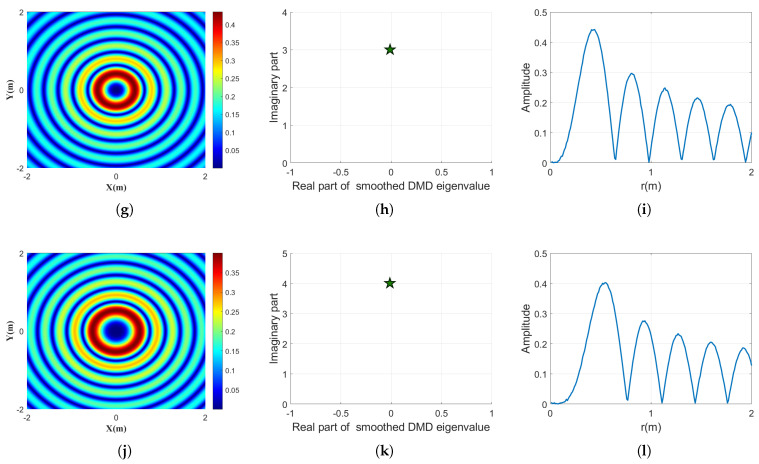
Illustration of the demultiplexing results for single Bessel beams with topological charges n=1,2,3,4 using the proposed smoothed DMD method. Subfigures (**a**,**d**,**g**,**j**) show the intensity distributions of the Bessel beams with topological charges n=1,2,3,4, respectively. Subfigures (**b**,**e**,**h**,**k**) present the corresponding eigenvalues in the complex plane extracted by smoothed DMD, where the imaginary part encodes the topological charge. Subfigures (**c**,**f**,**i**,**l**) display the recovered amplitude profiles of the extracted modes.

The corresponding results are illustrated in Figure 1. The left column shows the intensity distributions of the input Bessel beams in the xoy plane. As the topological charge *n* increases, the central dark core of the beam expands noticeably. This behavior reflects the inherent divergence of Bessel beams carrying higher-order OAM modes, where the increased azimuthal phase winding leads to broader spatial distributions. The middle column presents the dominant eigenvalues extracted by smoothed DMD in the complex plane. The imaginary parts of the eigenvalues increase proportionally with *n*, demonstrating that smoothed DMD successfully captures the OAM-dependent phase rotation encoded in the beam. The right column displays the radial amplitude distributions reconstructed from the extracted modes. The number and location of the oscillatory lobes agree well with the theoretical structure of Bessel beams, further verifying the validity of the demultiplexed results. These findings confirm that the smoothed DMD method is capable of accurately identifying and characterizing single-mode OAM beams, even under realistic conditions.

### 3.2. Mixed Bessel Beam

We further evaluate the performance of the proposed smoothed DMD method in separating mixed Bessel beams composed of multiple OAM modes. Two representative test cases are considered: one comprising topological charges n=1 and n=3, and the other comprising n=2 and n=4.

**Figure 2:**
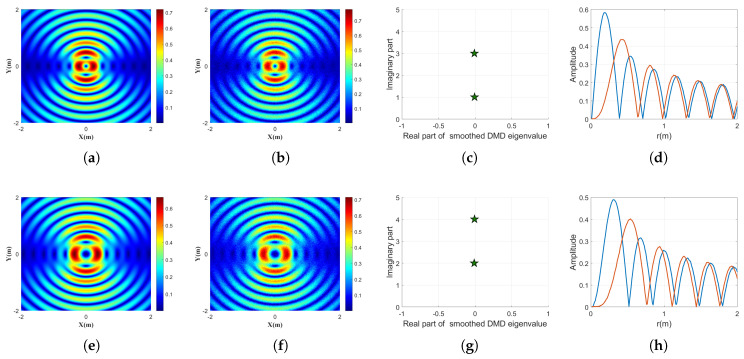
Demultiplexing results for mixed Bessel beams composed of two OAM modes, using the proposed smoothed DMD method. (**a**) Intensity distribution of the input beam composed of topological charges n=1 and n=3. (**b**) Signal in (**a**) with an SNR of 30 dB. (**c**) Extracted smoothed DMD eigenvalues corresponding to the mixed mode in (**b**), where the two distinct imaginary parts reflect the correct OAM components. (**d**) Extracted radial amplitude profiles for the two identified modes in (**c**), shown in different colors. (**e**) Intensity distribution of the input beam composed of topological charges n=2 and n=4. (**f**) Signal in (**e**) with an SNR of 30 dB. (**g**) Extracted smoothed DMD eigenvalues corresponding to the mixed mode in (**f**), again revealing two dominant modes with distinct imaginary parts. (**h**) Extracted radial amplitude profiles for the two identified modes in (**g**), demonstrating successful separation of both components.

Figure 2 presents the corresponding results. The left column shows the composite intensity distributions of the mixed beams. In both cases, the interference between different OAM modes leads to complex spatial patterns, deviating significantly from the clean concentric rings seen in single-mode beams. These patterns exhibit asymmetries and side lobes, making direct visual interpretation of the constituent modes difficult. The middle column displays the eigenvalues extracted using smoothed DMD. In both cases, two dominant eigenvalues appear along the imaginary axis, corresponding precisely to the theoretical topological charges of the input modes. This demonstrates that smoothed DMD is capable of correctly identifying the presence and strength of multiple OAM components, even when they are spatially entangled. The right column presents the reconstructed radial amplitude profiles for the extracted modes. The two profiles in each plot correspond to the separated contributions from the respective OAM topological charges. The number and positions of radial lobes are consistent with those expected from the individual Bessel functions of orders n=1,3 and n=2,4, respectively, confirming the successful decomposition of the mixed field.

**Figure 3:**
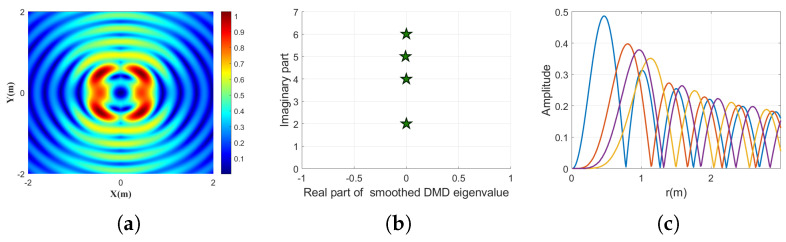
Demultiplexing results for mixed Bessel beams composed of four OAM modes, using the proposed smoothed DMD method. (**a**) Intensity distribution of the input beam composed of topological charges n=2, n=4, n=5, and n=6. (**b**) Extracted smoothed DMD eigenvalues corresponding to the mixed mode in (**a**), where the four distinct imaginary parts reflect the correct OAM components. (**c**) Extracted radial amplitude profiles for the four identified modes in (**b**), shown in different colors.

Figure 3 illustrates the demultiplexing of three overlapping Bessel beams carrying OAM topological charges n=2, n=4, n=5, and n=6 using the proposed smoothed DMD method. Figure 3a shows the initial intensity distribution of the mixed field, where the superposition of four OAM components produces a complex interference pattern. Figure 3b presents the extracted smoothed DMD eigenvalues, in which four distinct imaginary parts correspond precisely to the four OAM modes. Figure 3c displays the reconstructed radial amplitude profiles of each identified mode, clearly revealing their different propagation characteristics and validating the method’s ability to separate both the topological phase and amplitude distributions of the individual OAM components under noisy conditions.

These results demonstrate that smoothed DMD is not limited to single-mode detection but is also highly effective in the demultiplexing of multiple co-propagating Bessel beams. The method reliably recovers both the modal content and spatial structure, further supporting its applicability to practical OAM-based communication and sensing scenarios involving complex wavefronts.

### 3.3. Partial Aperture Receiving Scheme

**Figure 4:**
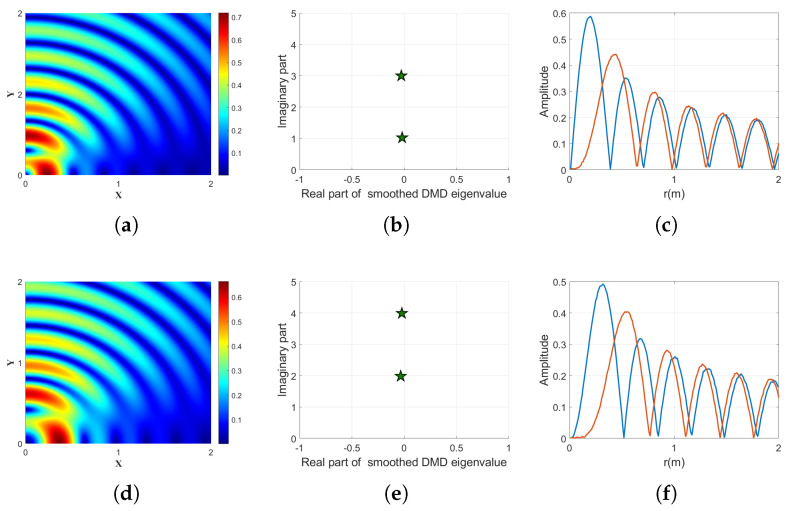
Demultiplexing results for mixed Bessel beams composed of two OAM modes with the partial aperture receiving scheme. (**a**) Intensity distribution of the input beam composed of topological charges n=1 and n=3. (**b**) Extracted smoothed DMD eigenvalues corresponding to the mixed mode in (**a**), where the two distinct imaginary parts reflect the correct OAM components. (**c**) Extracted radial amplitude profiles for the two identified modes in (**b**), shown in different colors. (**d**) Intensity distribution of the input beam composed of topological charges n=2 and n=4. (**e**) Extracted smoothed DMD eigenvalues corresponding to the mixed mode in (**d**), again revealing two dominant modes with distinct imaginary parts. (**f**) Extracted radial amplitude profiles for the two identified modes in (**e**), demonstrating successful separation of both components.

Figure 4 illustrates the performance of the proposed smoothed DMD method using a partial aperture receiving scheme, where the receiving array only captures a limited portion of the Bessel beamfront, mimicking realistic scenarios where full-aperture detection is not feasible due to physical or energy constraints. Two mixed OAM beams are considered: one composed of topological charges n=1 and n=3 (top row), and the other of n=2 and n=4 (bottom row). As shown in Figure 3d and Figure 4a, the intensity distributions of the input beams display strong divergence patterns characteristic of high-order Bessel beams.

Despite the partial aperture and the associated spatial truncation effects, the smoothed DMD method successfully identifies the two dominant OAM components in each case, as is evident from the extracted eigenvalues in Figure 3b,e. The distinct imaginary parts clearly correspond to the topological charges of the respective OAM modes. Figure 3c,f further validate the effectiveness of the method, where the reconstructed radial amplitude profiles for the demultiplexed modes show excellent separation and structural consistency with the expected modal patterns.

These results confirm that the proposed smoothed DMD method not only performs well under additive noise, but also exhibits strong robustness to spatial distortion caused by partial aperture detection and beam divergence. This highlights the method’s practical value in real-world scenarios, such as long-distance wireless communication, where complete field sampling is often unattainable.

### 3.4. Robustness Performance

To evaluate the performance of the proposed smoothed DMD method for OAM demultiplexing under Gaussian white noise, we simulate a scenario in which the input beam is a mixture of Bessel beams carrying two OAM modes with topological charges n=2 and n=4. Gaussian white noise is added at four different SNR levels: 3 dB, 5 dB, 10 dB, and 15 dB. Each configuration is tested using 500 Monte Carlo trials.

**Figure 5:**
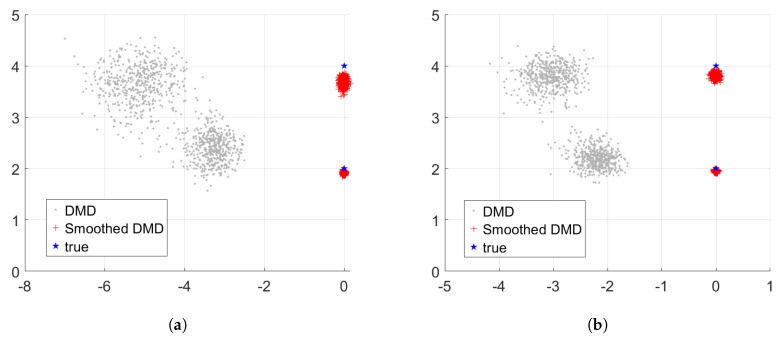

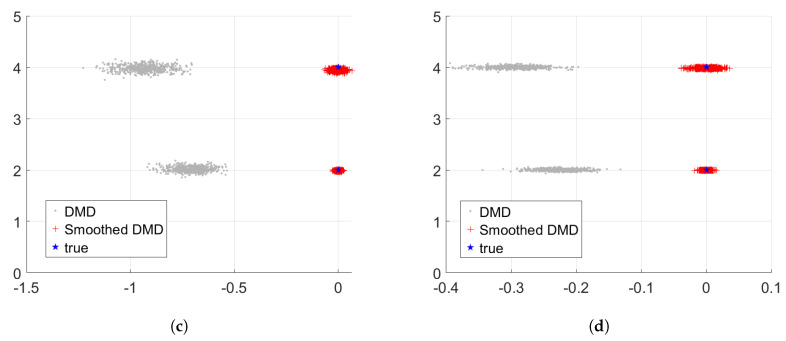
Comparison of demultiplexing results for mixed Bessel beams composed of two OAM modes between DMD and the proposed smoothed DMD methods in the presence of different Gaussian white noise environments: SNR = (**a**) 3 dB, (**b**) 5 dB, (**c**) 10 dB, (**d**) 15 dB.

**Figure 6:**
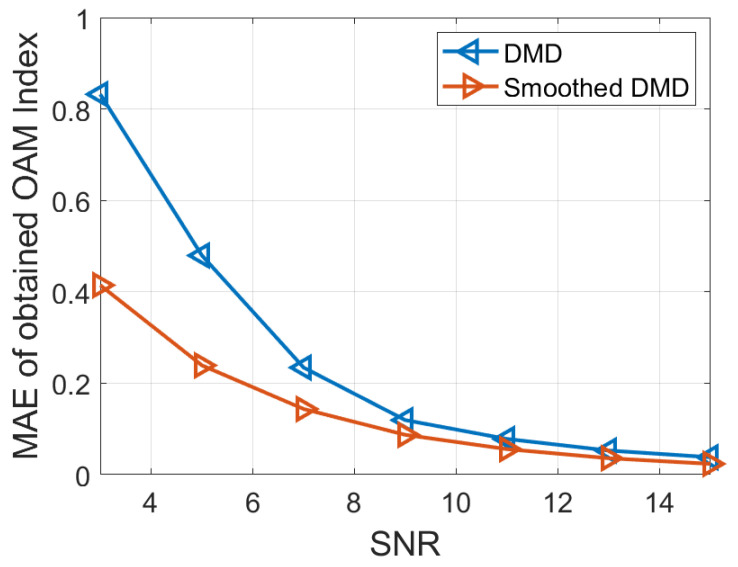
Comparison of MAE of obtained OAM index results for mixed Bessel beams composed of two OAM modes between the DMD and the proposed smoothed DMD method.

Figure 5 compares the demultiplexing results obtained by the conventional DMD and the proposed smoothed DMD methods under these noise conditions. The extracted eigenvalues are complex numbers, where the imaginary part represents the frequencies and the real part corresponds to the damping factors. To provide a more quantitative assessment, Figure 6 presents the mean absolute error (MAE) of the estimated frequencies for both methods across the four SNR levels. The results show that the accuracy of topological charge estimation improves for both methods as the noise level decreases, which is consistent with theoretical expectations. More importantly, the proposed smoothed DMD method consistently achieves lower MAE values across all SNR levels, indicating superior robustness and accuracy compared to conventional DMD. In particular, under low-SNR conditions (e.g., 3 dB and 5 dB), the conventional DMD method exhibits significant estimation errors and often fails to recover accurate topological charges. In contrast, the proposed method remains robust, demonstrating reliable performance even in highly noisy environments.

These findings confirm that the incorporation of a moving average step in the proposed smoothed DMD framework effectively suppresses random fluctuations induced by Gaussian white noise, leading to significantly improved estimation performance. Overall, the proposed method offers enhanced robustness and accuracy for OAM demultiplexing, particularly in low-SNR scenarios, making it a reliable solution for practical applications in noisy environments.

## 4. Conclusions

To sum up, we proposed a robust and noise-resilient demultiplexing method for electromagnetic Bessel beams carrying OAM, based on a smoothed DMD framework. By integrating a moving average pre-processing step with conventional DMD, the method significantly enhances the extraction accuracy of OAM topological charges in low-SNR environments. Numerical evaluations demonstrate that the proposed approach outperforms standard DMD in terms of both estimation accuracy and robustness against Gaussian white noise. Furthermore, the method operates effectively under practical constraints such as partial aperture detection and mode non-orthogonality, which are commonly encountered in real-world scenarios. These advantages make smoothed DMD a promising tool for OAM-based applications in wireless communication, sensing, and beyond.

## Data Availability

The raw data supporting the conclusions of this article will be made available by the authors on request.
